# Mapping the impact of generative AI in higher education: a scoping review of psychological and equity dimensions

**DOI:** 10.3389/fpsyg.2026.1856854

**Published:** 2026-06-08

**Authors:** Weina Ni, Jing Li

**Affiliations:** 1 School of Open Learning and Education, East China Normal University, Shanghai, China; 2 School of Management and Economics, University of Electronic Science and Technology of China, Chengdu, China

**Keywords:** cognitive offloading, digital divide, equity, generative AI, higher education, psychology, psychosocial-equity nexus, scoping review

## Abstract

The rapid integration of generative artificial intelligence (GenAI) into higher education has prompted growing scholarly attention toward its pedagogical and institutional implications. However, existing research has predominantly addressed technical capabilities and academic integrity concerns, while the psychological and equity dimensions remain fragmented and underexplored. This scoping review, conducted in accordance with PRISMA-ScR guidelines, systematically maps and synthesizes 87 studies published from January 2019 to March 2026 across six major databases to examine how GenAI influences learners’ cognitive and emotional processes and how its adoption intersects with educational inequalities. The findings identify five psychological themes—cognitive offloading, AI self-efficacy, emotional responses, identity threat, and trust—and six equity dimensions—digital divide, disability access, first-language inequity, socioeconomic stratification, gender and race disparities, and Global South constraints. Critically, only 7% of the reviewed studies explicitly examine the intersection of psychological and equity dimensions, revealing a significant gap in the literature. To address this fragmentation, the review introduces the Psychosocial-Equity Nexus, a novel three-layered recursive framework that integrates equity stratifiers, psychological states, and educational outcomes to conceptualize how structural inequities may be associated with psychological responses that, under certain institutional conditions, may contribute to the persistence or widening of educational disparities. The review offers actionable implications for research, institutional practice, and policy toward equitable and psychologically informed GenAI adoption in higher education.

## 1 Introduction

The advent of Generative Artificial Intelligence (GenAI), and especially ChatGPT, released towards the end of 2022, has brought about a paradigm shift in the domain of higher education (HE). Whereas the previous technological interventions used in education focused mainly on aiding the distribution of information, GenAI possesses the capacity to create human text, code, images, and analytical results in real time. This is a paradigm shift in the creation, access, and evaluation of knowledge within the academic sphere (Chavez et al., 2023; Eysenbach, 2023). As a consequence, GenAI is currently not only a supplementary technological resource, but it has become a disrupting agent that is turning everything in its wake upside down when it comes to pedagogic approaches and academic integrity. Current literature points out the rapid integration of GenAI technology into the academic environment globally, particularly the widespread use of large language models (LLMs). Uses of this technology have been extended to such practices as academic writing, content generation, tutoring, and research support (Baig and Yadegaridehkordi, 2024; Wang et al., 2025). These institutional reactions also show that there is a more comprehensive understanding of the importance of GenAI as institutions are developing policies to guide the usage of GenAI in teaching and assessment contexts (Wang et al., 2024; Jin et al., 2025). The popularity of the technology shows the importance of GenAI for the development of higher education today and indicates that many students are already employing these technologies in their studies (Shi et al., 2026). In essence, the psychosocial and equity impact of generative artificial intelligence in higher education are the topics of interest in the current review. The current review does not address issues related to curriculum development, institutional management, and performance of the technologies. The focus of the review lies in the ways in which the utilization of GenAI affects the psychological experiences of both teachers and learners through such constructs as cognitive offloading, self-efficacy, anxiety, trust, and identity perceptions in relation to inequalities in digital access, linguistic ability, disabilities, socioeconomic factors, gender, race, and Global South constraints of higher education (Hon, 2026).

Early scholarly debates about GenAI in higher education have largely centered on academic integrity, plagiarism, authorship, assessment validity, and institutional governance, particularly after the rapid adoption of ChatGPT and large language models in academic settings (Chiu et al., 2022; Crawford et al., 2023; Cotton et al., 2024; Farrokhnia et al., 2023; Sweeney, 2023; Jarrah et al., 2023). The capability of AI systems to complete complex academic assignments, including passing professional exams, and the problem of plagiarism, authorship, and assessment validity have been studied (Chiu et al., 2022). Simultaneously, it has been questioned how AI can be used ethically, how it can be governed, and how it requires institutional leadership to foster responsible adoption of AI technologies in learning institutions (Crawford et al., 2023). Although these views can be used to gain insight into the functional and regulatory aspects of GenAI, they are restricted in scope. In particular, the current literature has not focused much on two important aspects, which are paramount to comprehending the larger implications of GenAI in higher education, which are psychological effects and equity implications. Though there are studies that recognize the advantages and issues related to the introduction of AI, there is little synthesis of the impact GenAI can have on the cognitive and emotional processes of learners and how its implementation can contribute to or alleviate the available educational disparities. Such dimensions are especially crucial since they define whether GenAI can be used as an instrument in improving the learning experiences or whether it leads to novel types of stratifications within higher education systems.

This review is a structured mapping of an emerging evidence base rather than a definitive model-building exercise. Even though the Psychosocial-Equity Nexus is based on some repeating patterns that were identified in the included studies, the fact that only few explicitly intersectional papers were found implies that the framework is still tentative and needs to be empirically validated. Its worth is in its provision of a theoretically informed synthesis that can be used to guide future longitudinal, comparative, and intervention based research in more diverse higher education settings.

### 1.1 The psychological dimension

Generative artificial intelligence (GenAI) has an enormous impact on cognitive and psychological processes, particularly, it is raising the application of AI-assisted learning. The advent of such tools as ChatGPT has raised new concerns of academic integrity that has an impact on how students approach learning activities and assessment. Continuing on this, Farrokhnia et al. (2023) note the opportunities and threats of GenAI and say that it helps to make people more efficient and can make people too dependent and cause them to lose independent thinking. Similarly, the use of AI-generated content in the issues of contract cheating that could weaken the genuine learning process of students is also explained by Sweeney (2023). On the other hand, Jarrah et al. (2023) allege that the application of AI in academic writing is not only multifaceted and context-specific but also has an additional impact on the cognitive strategies of students in knowledge creation.

Regarding behavior and learning, Črček and Patekar (2023) document that students actively use ChatGPT to write, generate ideas and solve problems, and frequently use it as a main academic support resource. Similarly, Ngo (2023) concludes that the perceptions of students towards GenAI are both increased confidence and efficiency and dependency issues and decreased skill development. Rawas (2023) also mentions that GenAI can make learners more empowered as it can facilitate continuous and self-directed learning, but it can also change the old learning practices. To elaborate on this, Susnjak (2023) explicates that AI systems are moving the learning processes to automated and explainable models that may not require the deep analytical thinking. Also, Dehouche (2021) expresses worries regarding the misuse of generative models in academia, especially in plagiarism and ethical aspects. Lastly, Floridi and Chiriatti (2020) contend that the advanced AI systems have a fundamental impact on the human cognition and decision-making, which supports the notion that GenAI is not just a tool but a transformative phenomenon that will shape the way students think, learn, and engage with knowledge.

### 1.2 The equity dimension

GenAI also has the potential to exacerbate the phenomenon of inequalities within higher education, turning the already existing digital gap into the emerging AI gap. It should not be measured solely in terms of access to devices and the internet but rather also the differences in access to quality GenAI service, prompt literacy and institutional inequality. According to Dale (2021), there is an issue with the equitable distribution of access to the proper use of sophisticated artificial intelligence programs. Further, as highlighted by Elkins and Chun (2020), AI-generated texts are also capable of reproducing linguistic and cultural biases that benefit the dominant group. Following, according to Grassini (2023), technological infrastructure and readiness are some of the factors that impact the degree to which students can leverage GenAI technology. Within the field of education, for example, Mizumoto and Eguchi (2023) highlight that second-language English-speaking individuals might experience AI-facilitated bias against the dominant culture. Similarly, Leng (2024) points out that it is quite challenging to incorporate GenAI into specialized educational contexts.

Regarding accessibility and institutional barriers, according to Mogali (2024), the introduction of GenAI instruments in the educational sector could lead to an unequal learning process depending on the supporting infrastructure. Similarly, according to Kim et al. (2023), the acceptance and adoption of large language models depend on the institution, creating inequalities concerning access and usage. Westphaln et al. (2021) also mention some methodological factors which emphasize the necessity of conducting inclusive and systematic evaluation of the technologies under discussion. As for the definition of the Global South in this review, it is used to denote countries and regions which are frequently marginalized from the global knowledge generation due to uneven financial allocations, poor digital infrastructure, restricted institutional access to technology, and reliance on technology developed in high-income, English-speaking nations. This definition is analytical, not geographical, since inequalities can be observed even within high-income countries, and the Global South is highly heterogeneous (Currie et al., 2023).

### 1.3 The gap: why psychology and equity must be integrated

Importantly, the equity and psychological aspects of higher learning GenAI are not separate but closely interconnected. The institutional support, technological access, and language fluency determine the psychological reaction of a student to GenAI, e.g., trust, self-efficacy, or anxieties. As an example, the differences in technological preparedness and AI abilities impact the ways learners use and take advantage of AI systems (Min et al., 2023; Martínez-Plumed et al., 2021). In the same way, the variations in readiness and exposure to GenAI tools by educators and students also emphasize the differences in confidence, perception, and effective use (Kohnke et al., 2023; Bernabei et al., 2023). Simultaneously, the progress in the implementation of AI into knowledge-building processes indicates that the benefits of technology are context- and structure-dependent, and not universal (Chen B. et al., 2023).

Though there are these interdependencies, the current literature is still fragmented. General surveys of artificial intelligence in education are more likely to consider technological advances and uses without addressing the psychological or equity implications in a systematic manner (Chen et al., 2020). The literature addressing AI ethics and governance, e.g., Meskó and Topol (2023) and Chan (2023), highlights regulatory and ethical issues but fails to explicitly consider how ethics and regulation are applied in the context of higher education learning experiences. Also, the studies of natural language processing and transformer-like systems emphasize technical developments (Nassiri and Akhloufi, 2023), but do not focus on their dissimilar influence on various student groups. Although applied research examining GenAI in the educational context, such as writing performance and academic application (Alkamel and Alwagieh, 2024), tend to consider outcomes without incorporating psychological and equity lenses.

Consequently, both psychological and equity aspects of GenAI in higher education as a coherent system have not been studied in a systematic or scoping review. This disparity restricts the possibility to comprehensively learn how GenAI influences learning experiences and educational outcomes in various settings. Thus, the following research questions are discussed in this review

- RQ1: What are the psychological aspects of the use of GenAI in higher education that have been researched, and what are the key results?
- RQ2: What are the equity aspects of GenAI applied to higher education and what are the key outcomes?
- RQ3: What are patterns of interaction between psychological and equity dimensions that appear in the literature and what integrative framework do these patterns imply future research?

There are three main contributions made by this systematic scoping review. Firstly, it synthesizes the existing psychological and equity-related evidence pertaining to GenAI in higher education under one analytical framework. Secondly, there is a clear gap identified in the research conducted thus far, in that only 6 out of 87 articles have explicitly explored the relationship between the psychological and equity aspects. Thirdly, the Psychosocial-Equity Nexus is proposed as a novel framework to explore recurring themes found in the literature. It should be noted that this framework does not imply any causation between variables but serves merely as a basis for future research.

## 2 Methods

The scoping review methodology applied in this paper was to identify the existing literature on psychological and equity concerns regarding Generative Artificial Intelligence (GenAI) within higher education institutions. This scoping review has been carried out in accordance with the standards set out by the Preferred Reporting Items for Systematic Reviews and Meta-Analyses extension for Scoping Reviews (PRISMA-ScR) (Page et al., 2021).

### 2.1 Paradigmatic positioning

This review is based on the pragmatic-interpretivist paradigm. The pragmatist nature of the study is represented by means of a scoping review, which aims at systematically determining the limits, scope, and nature of scholarly research dealing with GenAI and higher education. The interpretivist nature of this research is expressed by the thematic analysis of psychological and equity-related aspects in different papers. Such an approach is reasonable for this particular research since it was not intended to test any hypotheses regarding causality. Instead, this research aimed to systematize disparate information, establish some recurring topics, and generate an initial theoretical framework explaining the connection between psychological aspects and equity-related issues in higher education made possible by GenAI.

### 2.2 Search strategy

There were six main academic databases considered [Scopus, Web of Science (Core Collection), ERIC (Proquest), PsycINFO (EBSCO), ACM Digital Library, and arXiv (preprints)]. It was important to choose these particular databases because they have interdisciplinary coverage, including a variety of educational, psychological, artificial intelligence, and computer science related articles.

The search strategy was structured in three Boolean blocks to enhance transparency, replicability and coverage of concepts. The initial block has captured GenAI-related words:

(“generative artificial intelligence” OR “generative AI” OR GenAI OR “large language model” OR LLM OR ChatGPT OR GPT OR Gemini OR Claude OR “DALL-E” OR Midjourney OR “multimodal AI”)*.

The second block captured higher education contexts:

(“higher education” OR universit OR college OR “tertiary education” OR postsecondary OR “post-secondary” OR undergraduate* OR postgraduate* OR graduate* OR “academic institution*” OR “university student*” OR “faculty member*” OR lecturer* OR instructor*)**.

The third block captured psychological and equity-related dimensions:

(psycholog OR cognitive OR metacognitive OR emotion OR anxiety OR trust OR “self-efficacy” OR identity OR “cognitive load” OR “cognitive offloading” OR “digital divide” OR equity OR inequality OR inclusion OR access* OR socioeconomic OR disability OR “first language” OR multilingual OR “non-native” OR gender OR race OR ethnicity OR “Global South”)**.

These blocks were joined with the help of the Boolean operator AND and terms in each block were joined with the help of the Boolean operator OR. The syntax requirements of each database (such as Scopus, Web of Science Core Collection, ERIC, PsycINFO, ACM Digital Library, and arXiv) were slightly adjusted by modifying search strings to fit the syntax of each database.

The search was limited to those studies that were published between January 2019 and March 2026. The search endpoint used to conduct the review was March 2026 as the review was conducted in the first quarter of the 2026 publication year.

The final search was conducted in March 2026. The search strategy was adapted to the syntax used by each of the databases while retaining the three conceptual blocks used throughout the search: terminology pertaining to GenAI, terminology regarding higher education, and terminology related to psychology and/or equity. All database results were exported to a reference management document and then reviewed for duplicates. Database-specific variations include the use of truncation, phrase searching, and limiting by field, wherever applicable. As the search interfaces in Scopus, Web of Science, ERIC, PsycINFO, ACM Digital Library, and arXiv differ, the actual syntax varied but maintained the same meaning across platforms. No additional weight was assigned to studies in the search process.

### 2.3 Eligibility criteria

Inclusion and exclusion criteria were pre-defined to make sure that only relevant studies were chosen and that the methodology was rigorous.

Inclusion criteria were: (i) empirical (quantitative, qualitative, or mixed-methods) and theoretical or conceptual articles; (ii) research on the application of the generative artificial intelligence (GenAI) to the context of higher education (including students and faculty); (iii) articles that explicitly covered at least one psychological dimension (quantity of cognitive, emotional, or behavioral outcomes) or one equity-related dimension (digital divide, disability access, language inequity, socioeconomic status, gender, race, or Global South contexts).

The exclusion criteria were: (i) articles on K-12 education or workplace learning but without specific emphasis on generative AI tools; (ii) articles on purely technical articles on model development, but with no focus on generative AI tools; (iii) articles that were on non-generative AI technologies, such as predictive analytics, or recommender systems, without having any focus on generative AI tools; (iv) articles that were on non-generative AI technologies, such as predictive analytics, or recommender systems, without having any focus on generative AI tools.

### 2.4 Screening and selection

To achieve reliability and reduce bias, the screening process was carried out in two phases by two reviewers who were independent of each other. First, titles and abstracts were filtered (Cohen 0.86), then full-text eligibility of studies was performed (Cohen 0.91). Any differences between reviewers were discussed and resolved by consensus with a third reviewer being consulted as needed. The entire selection process is represented with the help of a PRISMA flow diagram.

**Figure 1:**
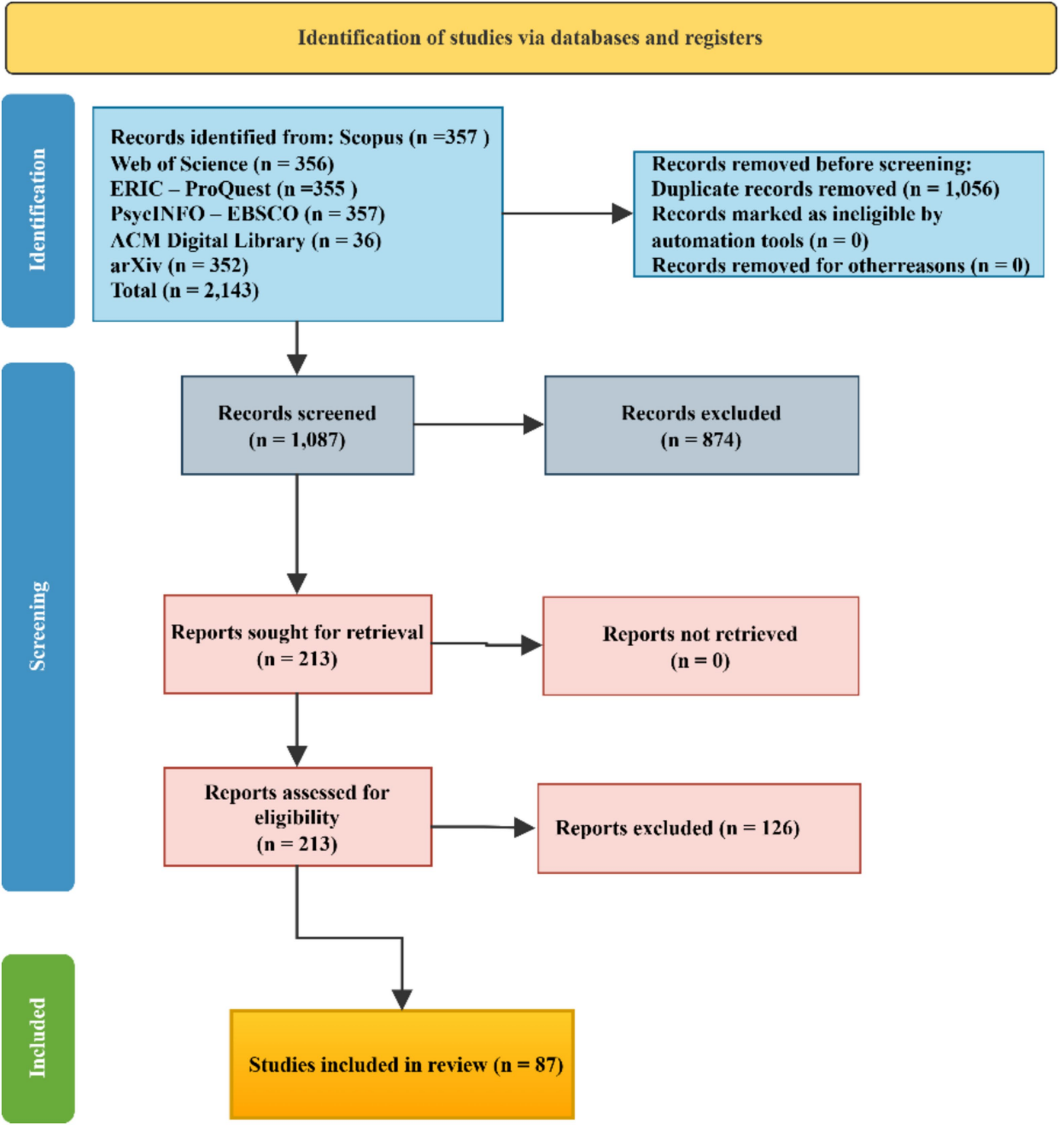
Study selection process flow diagram that will show the identification, screening, eligibility evaluation, and ultimate inclusion of studies in the review.

Figure 1 presents the PRISMA-ScR study selection process used in this review. Initially, 2,143 records were identified across the selected databases. After removing 1,056 duplicate records, 1,087 records remained for title and abstract screening. During screening, 874 records were excluded based on relevance and eligibility criteria. A total of 213 full-text reports were then assessed for eligibility, of which 126 were excluded for not meeting the inclusion criteria. Finally, 87 studies were included in the scoping review.

### 2.5 Quality appraisal position

In keeping with the objectives of a scoping review, this study did not perform any formal bias risk assessment or analysis on the quality of the selected studies. Rather than estimating the effect of interventions or making judgments about the quality of evidence, it sought to determine the scope, extent, and themes covered by the emerging body of literature. Nonetheless, one must acknowledge that failure to conduct any formal quality assessment remains a major weakness in this study. This is because studies of varying methodologies, such as cross-sectional survey studies, conceptual analyses, qualitative research, and mixed-method approaches, were considered as equals for purposes of synthesis.

### 2.6 Data extraction

A standardized framework was adopted for data extraction from all the studies selected for this review. The steps followed during the data extraction process were as follows: Author(s); Year of Publication; Location/Country; Type of Study; Sample; GenAI tools Used; Psychological Dimensions Investigated; Equity Dimensions investigated; Findings; Relationship between the two dimensions.

### 2.7 Data synthesis

The mixed-methods approach employed for the synthesis of evidence involves frequency and distribution calculations in the quantitative aspect and inductive-deductive analysis in the qualitative aspect. Specifically, frequencies of different types of studies based on their characteristics, psychological themes, equity themes, and studies that explicitly incorporate both the psychological and equity dimensions will be computed. This method was used solely for descriptive purposes and does not imply any evidence of effect size or causal relationship.

In qualitative synthesis, an inductive-deductive thematic analysis approach was applied to the identified relevant studies. For the inductive process, a deductive framework was built based on the questions raised and the literature review of GenAI, educational psychology, digital inequality, and ethical implications of AI use. Initial code sets include cognitive load, trust, self-efficacy, digital access, linguistic inequity, inclusion of people with disabilities, and socioeconomic inequalities. For the next step, inductive coding was carried out among the findings, discussion, and conclusions of the selected articles to reveal additional emerging themes.

In order to enhance the reliability and validity of our categories, comparisons and discussions among the reviewers helped refine our coding. For example, codes that showed up more than once or had relevance to the review questions were compared, combined, and classified under higher-level themes. Psychological themes were those that concerned cognitive, affective, behavioral, or identity-based reactions to GenAI. Equity themes were those that related to inequities associated with GenAI access, benefit, bias, exclusion, or other structural barriers. Finally, themes were classified as being integrated when a study mentioned an interaction between at least one equity condition and a psychological reaction or educational behavior.

The result was five psychological themes, six equity themes, and some number of studies on integration of psychology and equity issues. Lastly, an intersecting mapping exercise led us to categorize the articles we reviewed according to three types: psychology-only, equity-only, and psychology–equity integrated studies. Psychology-only studies focused on psychological reactions to GenAI and did not include any equity analysis. Equity-only studies explored GenAI use and its effects with no apparent psychological angle. The final category of integration was reserved for studies that considered interactions between equity conditions and psychological reactions. This classification helped distinguish the empirical patterns identified in the reviewed literature from the later conceptual interpretation developed in the Psychosocial–Equity Nexus framework.

## 3 Results

### 3.1 Study characteristics

The PRISMA-ScR process was used to select the study, which resulted in 87 included studies out of an original 2,143 records (1,087 after deduplication; 213 full texts evaluated). The temporal distribution shows that GenAI research is rapidly growing starting with 3 studies from January 2019 to December 2021, followed by 5 studies in 2022, 18 in 2023, 31 in 2024, and 30 from January 2025 to March 2026, which is a sign of a rapid increase in scholarly interest in the topic after the introduction of ChatGPT.

The literature is geographically concentrated in high-income areas, with North America (*n* = 34) and Europe (*n* = 22) leading, and Asia (*n* = 18), Australia/New Zealand (*n* = 8), and a small representation of the Global South (*n* = 5). Quantitative studies are the most prevalent (*n* = 41), then comes qualitative (*n* = 28), mixed methods (*n* = 12), and conceptual studies (*n* = 6). Regarding tools, research is extremely focused on ChatGPT and large language models (*n* = 79) and little on image-based (*n* = 5) and multimodal systems (*n* = 3). In general, the sphere is growing at a rapid pace but is still unequally distributed regionally and methodologically.

### 3.2 RQ1: psychological dimensions

The results indicate that there are five psychological themes, including cognitive offloading and metacognition, AI self-efficacy, emotional reactions, identity threat, and trust in AI outputs. One of the prevalent trends is cognitive offloading, where students employ GenAI to lessen mental load, which enhances efficiency but may undermine in-depth learning and memorization. Research shows that the dependency on AI improves the short-term performance at the cost of decreased autonomous cognitive processing (Patac and Patac, 2025; Deng et al., 2025; Youssef et al., 2024).

Other important variables to consider include AI self-efficacy. It was found by Shuhaiber et al. (2025) and Haq et al. (2025) to have a positive correlation with perceptions of usefulness and efficient usage of GenAI solutions. According to Verano-Tacoronte et al. (2025) and Deng et al. (2025), negative emotions experienced by educators and students are anxiety, although both efficiency and stress levels fall at the same time. Users’ trust in the output generated by AI solutions also depends on their level of expertise, and in accordance with Yeung et al. (2026), less skilled users tend to overestimate the quality of the work while more knowledgeable people prefer critical approaches. Issues related to identity also arise, as reported by Yilmaz and Karaoglan Yilmaz (2023): students lack confidence in their own academic merit when AI handles high-level cognitive processes.

### 3.3 Conclusion

The conclusion drawn from the review is that psychological literature tends to examine efficiency and user perceptions with no use of longitudinal evidence or interventions. The structured analysis of the identified themes can be explained by the presence of theoretical and methodological frameworks, such as those proposed by Arksey and O'Malley (2005). Meanwhile, the identification of AI usage patterns is also facilitated by an extended context of the digital learning environment described by Chen P. et al. (2023). Despite being a relatively new area of research, equity issues tend to remain underexplored in psychological studies.

### 3.4 RQ2: equity dimensions

Six overall equity themes were identified in the analysis: digital divide, disability access, first-language inequity, socioeconomic stratification, gender and race disparities, and Global South limitations. The most noticeable problem is the digital divide, in which disparities in access to sophisticated GenAI tools form a two-level system of learning. Blahopoulou and Ortiz-Bonnin (2025) indicate a significant difference in the attitude and benefits between users and non-users, and Uppal and Hajian (2025) demonstrate that the access to AI tools is a significant determinant of productivity and academic engagement. On the same note, Greitemeyer and Kastenmüller (2023) and Currie (2023) indicate that unequal access also has a bearing on academic integrity behaviours and ethical use.

The access to disability has both positive and negative results, with GenAI being personalized and access to it being a barrier. Cao et al. (2025) and Strzelecki (2024) show that institutional and technological support can be adopted differently whereas Habibi et al. (2023) show that usability and acceptance can be different among student groups. Notably, Patvardhan et al. (2024) demonstrate that GenAI can be used to facilitate individualized learning in students with disabilities, but accessibility standards are still important issues.

Another important dimension that is critical is first-language inequity where non-native English speakers are disadvantaged by language biases within AI systems. As Liang et al. (2023) affirm, AI detection tools are more likely to punish non-native writing style, whereas Jiang et al. (2024) affirm that automated assessment systems are biased, which further burdens non-native learners. These results imply that inequity in language is systematically embedded in GenAI systems.

Socioeconomic stratification also contributes to the increase in disparities, with access to high-quality AI tools, devices, and consistent internet being unequal. Research shows that low-income students are restrained in using advanced features, which restricts their academic benefits (Blahopoulou and Ortiz-Bonnin, 2025; Uppal and Hajian, 2025). Although less studied, gender and race differences reveal some trends, and it is possible to note that there are differences in AI usage, confidence, and bias in generated outputs (Greitemeyer and Kastenmüller, 2023). Lastly, the Global South is underrepresented, and the barrier of the structure, like a lack of infrastructure and a Western-based knowledge system, prevents an equal uptake.

On the one hand, digital access and usability issues prevail in equity research, whereas crucial aspects like gender, race, and Global South views are still vastly unexamined. The results indicate that GenAI does not only mirror existing inequalities but also poses a risk of increasing them when equitable access and inclusive design is not done in a systematic manner.

### 3.5 RQ3: intersection of psychological and equity dimensions

The combination of the psychological and equity aspects is the key contribution of this review. Out of the 87 studies that were used, 54 (62) studies were carried out on psychological aspects only, 27 (31) studies were carried out on equity issues only and only 6 studies (7) actually examined the interaction between the two dimensions. This disequilibrium underscores a severe disjointure in the literature. Recent findings demonstrate repeatedly that the equity factors are an important variable that can be used to moderate psychological responses to GenAI. In general, considering socioeconomic differences as a case in point, unequal access to AI self-efficacy where different levels of confidence and learning scores are related to unequal access proves existence of AI generation gap among the users (Chan and Lee, 2023; Babu et al., 2024). Similarly, the perception and interaction of the students with GenAI depends on the inequalities in the access and support systems of the context (Chan and Hu, 2023).

Intersectional results also indicate that psychological vulnerabilities are increased by structural inequities. In Global North and South comparisons, Olaniyan et al. (2026) depict that epistemic inequalities are influencing the development of trust, reliance, and perceived value of AI, which is biased towards Western knowledge systems. On the inclusion perspective, Becher and Kogan (2025) mention the importance of considering both structural access and individual experiences to be able to participate in higher education equally; Othman and Al Mutawaa (2023) also note the significance of accessibility in influencing cognitive engagement in students with disabilities. The psychological effects of language inequities based on the language also overlap with the work by Kwak and Pardos (2024) who show that multilingual imbalances in AI systems affect the confidence and usability of the system further supported by systemic biases present in the AI models Lee et al. (2024).

Recent research recommends the use of equity-conscious systems in the process of AI adoption to mitigate these compound effects. Mangal and Pardos (2024) introduce intersectionality-aware machine learning methods to address the issue of bias, whereas Bayer et al. (2024) show that equity-oriented analytics can help disclose the latent inequity in educational achievement. Also, Dakakni and Safa (2023) believe that issues of ethics and equity in the application of AI directly affect the perception of learners and their psychological reactions, which supports the importance of a comprehensive approach. In general, the evidence confirms that psychological experiences with GenAI are not comprehensible outside of equity conditions. Nevertheless, none of the studies combines the psychological conditions, equity statuses, and institutional policies in a single model. This lack highlights the need to have a systemic framework, including the proposed Psychosocial-Equity Nexus, that can help define the recursive and interdependent nature of these dynamics in higher education.

It is important to distinguish the empirical mapping from the conceptual interpretation. Empirically, the review found that only 6 of 87 studies explicitly connected psychological and equity dimensions. Theoretically, the Psychosocial–Equity Nexus interprets this limited but recurring evidence as suggesting possible links among structural conditions, psychological responses, and educational outcomes. Therefore, the framework should be treated as a synthesis-based proposition rather than a confirmed explanatory model.

## 4 Discussion

### 4.1 Summary of principal findings

The results indicate that GenAI research conducted in the higher education sector today is analytically fragmented into two separate spheres. Research in psychology reveals how individuals react both intellectually and emotionally to GenAI, whereas research on equity shows how factors such as access, language, disability, socio-economic background, and location influence engagement. Yet there is very little interaction between these two spheres. The gap prevents an understanding of why the same GenAI application causes one learner to be confident and supported in their studies and another learner to feel anxious or excluded.

### 4.2 Psychosocial–equity nexus

The Psychosocial – Equity Connection should be seen more as an interpretive framework than as an empirical model because it is based on patterns observed in the included literature, especially the relatively few papers that made connections between equity variables and psychological factors. Most of the included studies being either cross-sectional, subjective, or conceptual in nature, the framework does not make any statement about causality. Rather, it is meant to be used as a guide in organizing relationships for further empirical research.

**Figure 2:**
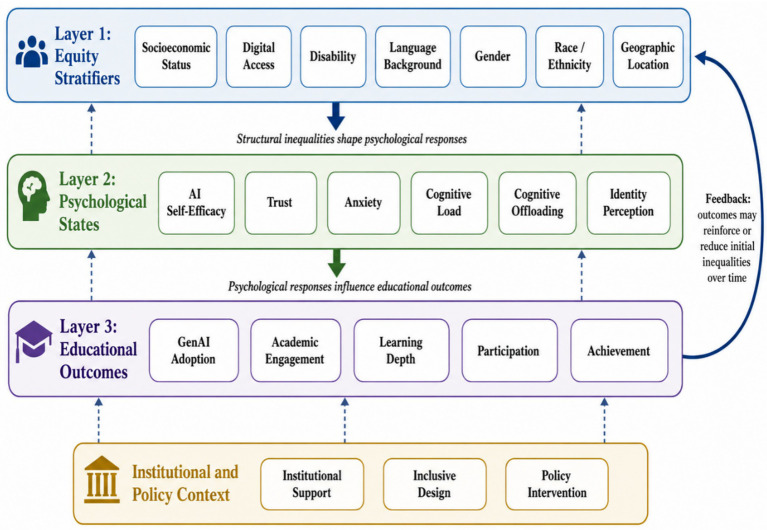
Three-layered psychosocial–equity nexus in GenAI-mediated higher education.

This review is a synthesis of the literature that suggests the Psychosocial Equity Nexus as a temporary, integrative framework that can describe the dynamic interplay between equity conditions and psychological processes in the formation of generative AI (GenAI) use in higher education (Babu et al., 2024). Learning experiences are viewed as the result of the interaction between the structural inequalities and the individual cognitive-affective reactions, but not of the individual technological or behavioral factors. It is based on the trends seen in the limited yet consistent body of intersectional literature found in this review and is meant to inform future empirical research and rather than to serve as a fully validated explanatory model (see Figure 2).

The framework comprises three layers that are interrelated. The layer 1 is the equity stratifiers, such as socialeconomic status, digital access, disability, language background, gender, race, and geographic location. The layer 2 is psychological states, such as AI self-efficacy, trust, anxiety, cognitive load, cognitive offloading, and identity perception. Layer 3 reflects educational results, such as the adoption of GenAI, academic activity, depth of learning, participation, and success. The model proposes that psychological reactions to GenAI may vary according to structural conditions such as access, language background, disability support, socioeconomic status, and institutional context. The feedback arrow suggests that the result of education can either reinforce or decrease the initial inequalities over time, based on whether or not these institutions support, are inclusive, or intervene in support of prevention.

The results attributed to RQ3 give a faint empirical evidence to support the proposed framework as only 6 out of 87 studies included in the results explicitly analyzed the interaction between psychological and equity dimensions. Thus, the Psychosocial-Equity Nexus must be approached as a tentative conceptual framework, and not a fully validated empirical model. Specifically, six combined studies reveal that equity conditions can determine the psychological reactions, which consequently affect educational behaviors. As an example, access differences, language ability, and disability accommodation seem to impact the AI self-efficacy, cognitive load, and disability accommodation, which, in turn, affect adoption behaviors and academic engagement.

According to Table 1, the six studies all lead to the conclusion that disparities that are related to equity are systematic in determining psychological experiences, which consequently affect learning practices and outcomes. Nevertheless, the fact that there are few such studies suggests that the intersection is significantly understudied. Thus, despite its logical synthesis of existing evidence, the recursive nature of the Psychosocial Equity Nexus can be viewed as hypothetically suggestive and in need of further empirical confirmation, especially in non-WEIRD and diverse settings of higher education.

**Table 1:** Empirical anchoring of the psychosocial–equity Nexus in integrated studies.

Study	Equity stratifier	Psychological state	Educational / behavioral outcome	Nexus layer linkage
Chan and Lee (2023)	Generational access / digital familiarity	AI self-efficacy, engagement	Higher adoption among Gen Z; differential confidence levels	Equity → Psychology → Adoption
Chan and Hu (2023)	Access and usage disparities	Perceived benefits vs. anxiety	Mixed attitudes influencing usage behavior	Equity ↔ Psychology
Olaniyan et al. (2026)	Global North vs. South	Epistemic confidence, trust	Knowledge translation dependency; epistemic inequality	Equity → Psychology → Knowledge production
Othman and Al Mutawaa (2023)	Disability / accessibility	Cognitive load, frustration	Uneven learning experience due to accessibility gaps	Equity → Psychology → Learning efficiency
Kwak and Pardos (2024)	Language (multilingual inequity)	Identity threat, cognitive burden	Reduced participation and confidence in non-native users	Equity → Psychology → Participation
Dakakni and Safa (2023)	Ethical and institutional inequity	Anxiety, technostress	Hesitation and avoidance behaviors in AI use	Equity → Psychology → Behavioral intention

### 4.3 Comparison with existing frameworks and novelty

The proposed Psychosocial Equity Nexus expands and combines the knowledge of a number of already established theoretical points of view and discusses their weaknesses with respect to generative artificial intelligence (GenAI) in higher education. First, the conventional models of digital divide have mostly conceptualized inequality based on access to technology (first-level divide) and, more recently, on skills and use (second-level divide). Although these models can be applied in identifying structural differences, they fail to sufficiently address the psychological impacts of unequal access, including low self-efficacy, increased anxiety, or identity threat, which this review has found to be important mediating processes (Pratama, 2025; Beckman et al., 2025). Second, the models, like the Technology Acceptance Model (TAM) focus on perceived usefulness and ease of use as predictors of technology adoption. Nevertheless, TAM mostly presupposes homogeneous users and does not explicitly consider inequalities based on socioeconomic status and language, as well as accessibility, and does not include the emotional or identity-based reactions to AI. Likewise, the cognitive load theory describes the way learners cope with mental strain when performing tasks but does not take into account how the cognitive processing is influenced by structural inequalities or unequal access to AI services.

The theory of capital as developed by Bourdieu offers a useful perspective on understanding inequality in the use of GenAI, based on the equity-oriented approach to inequality. The disparities in the access to high-quality tools, digital literacy, and linguistic fluency may be interpreted as digital, cultural, and linguistic capital, which benefits some groups and disfavors others (Memarian and Doleck, 2023). The Psychosocial Equity Nexus expands upon these by directly connecting these types of capital with psychological conditions, and showing how unequal allocation of resources can be translated into differences in confidence, trust, and cognitive interaction. Simultaneously, the technostress literature provides valuable information on emotional and behavioral effects of using technologies such as anxiety, overload, and avoidance behaviors (Kasneci et al., 2023; Jovic et al., 2025; Chan, 2023). These constructs are directly portrayed in the findings of this review about AI-related anxiety, frustration caused by hallucinations, and mistrust in outputs. But the current models of technostress generally consider stress as a personal-level reaction, but the new framework places these experiences in the context of more structural inequalities and the stress reactions are not equally distributed, but rather they are mediated by access, capability, and context.

In general, the Psychosocial-Equity Nexus is a valuable addition to the literature, as it helps to fill the gaps between structural and psychological approaches and provides a more in-depth view of how GenAI influences higher education learning. The framework combines equity stratifiers with cognitive, emotional and identity-based processes into a recursive system, thus shedding light on previous one-level and one-dimensional explanations and setting the stage of future multi-level and intersectional research (Strzelecki, 2023; Hornberger et al., 2023).

### 4.4 Limitations of the included studies

There are several limitations that need to be highlighted. First, no evaluation of methodological quality was carried out. While this aligns with the mapping intent of a scoping review, it affects the strength of the synthesis, since studies of various methodological qualities were used. This means that conclusions should be drawn from thematic patterns described by the literature.

Second, despite an exhaustive search carried out using six databases, some articles might have been missed. For example, the review only included English-language studies. In addition, not all grey literature and other technical databases were used, which may have caused publication and language biases. This could be particularly problematic given that GenAI research is advancing rapidly in many different areas.

Third, the majority of the included studies used cross-sectional, self-reporting, or conceptual methods. While useful for establishing perceptions, reporting experience, and identifying themes, such methods cannot establish causality. Consequently, the results regarding the relationships between the use of GenAI tools, psychological reactions, and equitable outcomes must be understood as associations or interpretations rather than causal links.

Fourth, the literature is geographically imbalanced and underrepresented by Global South cases and non-English-speaking learning contexts. The findings may not be generalized to institutions with distinct technology facilities, language backgrounds, and regulatory contexts.

Lastly, only a few studies specifically analyzed the interconnection between the psychological and equity components. It means that the framework of the Psychosocial-Equity Nexus should be treated as an experimental model for the structuring of future investigations rather than a scientific theory.

### 4.5 Contribution of the review

The current paper makes three main contributions. Firstly, it attempts to unify scattered literatures on the psychological and equity dimensions of GenAI in HEIs by considering both of them in one analytical framework. Secondly, it draws attention to the significant problem that only six out of 87 examined papers consider the intersection between psychological and equity dimensions. Thirdly, the current paper proposes a framework entitled “Psychosocial-Equity Nexus” which is seen as an attempt to analyze how structural inequalities affect psychological processes related to the adoption of GenAI technology and how these processes influence educational behavior, trust, confidence, and performance. Contrary to other frameworks that could be presented as theories, the authors present it only as a guide for future research.

## 5 Implications

Implications of this review extend far into the realm of academic investigation, implementation, and public policy-making. The most valuable study design for future research would be an intersectional one that simultaneously considers psychological and equity variables along with longitudinal and experimental designs for exploring potential temporal connections, mechanisms, and context-dependent relationships (Nasho Ah-Pine and Awuye, 2025; Annamalai and Nasor, 2025). Greater diversity in Global South contexts must be considered to increase generalizability and expand the focus from large language models to multimodal generative artificial intelligence models. Furthermore, future studies must consider whether there exist recursive feedback loops between equity situations, psychological conditions, and educational attainment in order to create a better understanding of their dynamics over time. Practically, to reduce inequities, institutions need to incorporate universal design principles into the implementation of AI tools, offer specific psychological assistance to students, create AI self-efficacy training, and implement language-inclusive assessment practices (Fidan and Gencel, 2022). Subsidized access to high-quality AI tools at policy level to reduce the AI divide, revised disability accommodation models such as GenAI and strategic investment in localized AI systems, especially in low-resource settings and Global South settings, are required to achieve equitable and inclusive adoption of AI (Klimova et al., 2023; Alamri et al., 2020).

## 6 Conclusion

The expansion of generative artificial intelligence in higher education is not only an instructional issue but also a psychological and equity-related concern. This scoping review indicates that current research has often examined psychological and equity dimensions as separate areas of inquiry. Psychological studies have mainly focused on learners’ cognitive and emotional responses to AI-mediated tasks, while equity-oriented studies have examined access, language, disability, socioeconomic position, and geographic context. Nevertheless, few studies have been carried out to study the intersection between these dimensions.

The proposed framework of the Psychosocial-Equity Nexus could be a tentative approach toward organizing such fragmentation by examining the intersection between social placement, psychological experience, and educational outcome. Rather than treating access, self-efficacy, and language bias as three separate independent variables, the nexus acknowledges their potential relationship to one another and the possibility of their coexistence. Yet since the Psychosocial-Equity Nexus itself is derived from only a few studies that are integrated, the nexus should be seen only as an exploratory model for future empirical studies.

Future research should move beyond a simple description of the intersection of those concepts. For instance, more studies might examine the impact of institutional intervention, inclusive design, and AI literacy training on overcoming institutional and psychological obstacles to the adoption of generative AI in higher education through longitudinal studies, comparative analysis, and experimental methods. Without any supporting data, any causal inference might be considered overly ambitious. This review, then, presents a structured synthesis and an exploratory framework for future empirical studies on the chosen topic.

## Data Availability

The original contributions presented in the study are included in the article/supplementary material, further inquiries can be directed to the corresponding author.
